# The evolving network of labor flows in the Stockholm Region: Sector dynamics, connectivity and stability

**DOI:** 10.1007/s41109-017-0056-x

**Published:** 2017-10-10

**Authors:** Hernan Mondani

**Affiliations:** Department of Sociology, Stockholm University, Stockholm, Sweden

**Keywords:** Interorganizational labor flows, Labor market dynamics, Sector dynamics, Network science, Network stability, Centrality, Assortativity, Network backbone overlap

## Abstract

Evolutionary theories of organizational change aim at finding processes that introduce structural variations in organizational variables and the conditions under which they can survive and be reproduced. However, the theory is limited by the lack of knowledge on interactions between organizations and the stability of interaction patterns over time. In this study, we use the network of interorganizational labor flows and tools and concepts from network science to inform the study of organizational evolution at the level of sector dynamics, in particular along the dimensions of connectivity and stability of labor flow patterns. We use a unique Swedish longitudinal register on employment in the Stockholm Region from 1990 to 2003. We find that the network is characterized by positive sector assortativity, and the public sector is relatively more tightly connected than the private one. A stability analysis shows that public organizations survive longer time in the dataset, and movements within publicly-owned organizations are the most stable while movements within the private sector are least stable. A network backbone overlap analysis shows that movements within the public sector are structurally stable over larger periods, while the ones within the private sector change quickly after a few years. We also find that the distributions for degree, interorganizational flows and betweenness centrality are highly skewed and “fat”-tailed; the public sector consistently has fatter tails than the private sector in all distributions. Implications for our understanding of how publicly and privately owned organizations are connected and react to external shocks are discussed.

## Introduction

### Organizational evolution and sector dynamics

The social world is structured by organizations. Our activities, decisions and a great deal of our actions take place within or are affected by various forms of organizations, from states and companies to voluntary associations (Ahrne 1994). Thus, it is not surprising that social scientists have long been interested in the processes by which organizations and organizational forms come to be, change and cease to exist.

Research on organizational change stretches across disciplines and encompasses various levels of aggregation, from studies of particular processes like team dynamics and decision making, through case studies of particular organizations, all the way up to the characterization of groups of organizations such as sectors and industries. We take our theoretical departure in the evolutionary approach to organizational change, originally formulated in Aldrich (1979) and more recently in Aldrich and Ruef (2006). Similar conceptual frameworks and process descriptions are found in evolutionary economics (Baum and Singh 1994; Nelson and Winter 1982), management science (Van de Ven and Poole 1995) and organizational psychology (Bartunek and Woodman 2015).

As Aldrich and Ruef (2006, ch.2) put it, the evolutionary approach is a meta-theory inspired by biological evolution and population ecology. It conceptualizes change in terms of four main processes: i) emergence of variation in organizational traits, ii) selection of traits that survive and are reproduced, iii) retention of these surviving traits, iv) competition among organizations in a changing environment.

We believe there is merit to the evolutionary approach, inasmuch as it is a process-oriented framework that focuses on how variations and new organizational patterns are created and sustained through competition and interaction. However, there are analytical difficulties with this approach. The main issue is that the four processes named above are coupled with each other and very difficult to disentangle, even theoretically. In order to get a better analytical grasp of the situation, we think it can be fruitful to look at common aspects underlying all organizational evolution. We focus on two aspects of organizational evolution: connectivity and stability.

On the one hand, organizations can interact in various ways, for instance, by being part of the same legal entity or conglomerate, or by competing in the same market. They can also be connected through the people that they employ. By *connectivity* we mean how the different parts of the organizational system are linked to each other by employee movements. ^1^ Are all organizations connected to each other? Can any organization be reached from any other? Is it more likely for public organizations to be linked to other public organizations? Describing how the connectivity structure looks like and how it evolves in time provides valuable insights as to how resources and information may circulate.

On the other hand, organizational evolution has much to do with the *stability* of organizational structures and processes, something that March (1981) refers to as stable processes of change. Are the organizations in the system the same over time? Does the pattern of change in organizational properties look alike for different sectors, and is it stable over time? And how is all of this affected by macroeconomic phenomena like crises? Stability can be a proxy for the retention process of the evolutionary approach.

In this article, we study two interacting populations of organizations: the public and the private sectors of the economy. This coupling is a relatively unexplored subject, since most studies in the literature on organizational change focus on the private sector. Within economics, the public sector has been studied from different perspectives (Gretschmann 1991). Although these perspectives differ from each other, there are some commonalities. Public activities and service provision are typically seen as more bureaucratic, inflexible and inefficient than market-driven private activities. Public services such as administration, transport, education and health, even when subject to restructuring and privatizations, require large bureaucracies to function and are difficult or impossible to displace. On the other hand, the private sector relies on the market logic for resource allocation, and certain private activities can be more flexible and less bounded to the territory.

### Exploring organizational evolution through the network of labor flows

Our analysis makes use of a network of labor flows to study connectivity and stability in organizational evolution. Networks provide a natural representation for the study of organizational processes (Borgatti and Foster 2003). There are different kinds of organizational networks. Some research looks into the dynamics of networks of employees between organizations, for example on how directory boards in different companies are interlocked (Levine 1972; Bohman 2012). There are also studies on networks of employees within organizations (Kilduff and Krackhardt 2008) focusing on questions of leadership, organizational culture, etc. (Uzzi et al. 2007). There are moreover networks between the organizations themselves, like in the case of buyer and sellers in a production system (Atalay et al. 2011), industrial sectors (McNerney et al. 2013), or world trade (Fagiolo et al. 2013), to name a few examples.

Of even more relevance to us are organizational networks created from the interplay between people and organizations. Classical examples include job search and matching through contact networks (Granovetter 1974) and the filling of vacant positions created by people changing organization (White 1969; 1970). Employee flows in the labor market fall within this class as well. As Collet and Hedström (2013) point out, such mobility networks put organizations in contact, and give both the arrived employee and her colleagues and employer the opportunity to acquire information on the employee’s previous organization.

In our study of interorganizational labor flows, the population of organizations is represented as a network where nodes are organizations and edges are movements of employees between organizations. A related study of labor flows with Finnish Longitudinal Employer-Employee Dynamics (FLEED) with a robustness analysis with Mexican company data can be found in Guerrero and Axtell (2013), who call it a *labor flow network*. The authors’ analyses are mainly based on undirected versions of the network, which makes our study richer in depth. The Finnish data has both public and private sector, but the article does not make an explicit distinction between them, unlike our case, and this is also a strength.

We can track organizations and individuals’ membership to organizations in the Stockholm Region over a period of 14 years and construct an evolving network. Particular attention is given to differences between movements within and between the public and private sectors, a type of interaction that is not frequently studied in a network context. This kind of analyses on the scale of a whole region would be impossible to carry out without these detailed longitudinal data, and as such, it is one of the contributions of our study. This article also contributes to the literature on organizational change by linking micro-level interorganizational movements with macro-level patterns at the population of organizations.

Furthermore, the data covers the period 1990 to 2003. This historical period in the Swedish context is deeply marked by the economic crisis of the 1990s. By 1993, unemployment in the country had risen to 8.3%. Welfare service provision in areas such as health retracted to historical minimums and the increase in social expenditure for unemployed contributed to a strong decrease in GDP only reversed from 1994 and onwards (Bergmark and Palme 2003). As the decade progressed, the economy recovered, at first through an increase in private service activities, but the full employment situation of previous years was never restored (Palme et al. 2002). Thus, this very particular macroeconomic setting gives us the opportunity to see how the movement structure changes over time during the initial years of the crisis— which is an external shock in the region— as well as during the recovery years thereafter.

### Aim of this study

The aim of this study is to use the network of interorganizational labor flows and tools and concepts from network science to inform the study of organizational evolution at the level of sector dynamics, in particular along the dimensions of connectivity and stability of labor flow patterns. We do so by using a unique longitudinal database on employment in the Stockholm Region for the period 1990-2003 allowing for traceability of individual interorganizational movements over time and across sectors.

## Data

### The database

We use the Stockholm database for our analyses. It is a compilation of Swedish governmental registers compiled by Statistics Sweden (*Statistiska centralbyrån*, SCB)^2^. It contains information on organizations in the Stockholm Region for the period 1990-2003. All organizations with employees registered in the region are included. Additionally, it is possible to use the legal ownership to distinguish between organizations belonging to the public and private sectors. Each organization has a unique, de-identified ID number that allows for longitudinal tracking across the whole period.

We also have information on the first source of employment for every individual 16 years old or older employed by an organization in the Region during the period. Longitudinal traceability of employees is possible through another set of de-identified unique IDs. By comparing the membership of a given individual between two consecutive years, we can determine if the person moved to a new organization or stayed. We measure the size of an organization at a given year as the sum of registered employees occupying positions in fixed workplaces at the end of that year. The fact that we sample only once per year means that we fail to observe employment movements within shorter periods. At the same time, it excludes variations due to seasonal job changes.

Throughout our analyses, we have tried to use a number of variables that keeps the analyses meaningful yet not too complex to handle. More variables are available in the data and not used here. For example, there is information on the industrial activity of the workplace (and by aggregation of the organization), for both public and private organizations, as well as information on the geographical area where the workplace is located. Additionally, there is occupational information in the form of occupational category for each individual employee.

To conclude, let us mention other studies that have used the same database. On the one hand, some studies deal with other research questions such as simulations of unemployment neighborhood effects among young people (Hedström 2005, ch.6) or dynamical systems modeling of school segregation in Stockholm over time (Spaiser et al. 2016). On the other hand, there are also some studies on questions related to our article, like the paper by Collet and Hedström (2013) on how mobility between workplaces can contribute to create individual-level social ties. The latter study adopts a micro-level perspective on the data, where the organizations are means for employees to form social acquaintance ties. Our approach, on the other hand, uses aggregated individual-level data to study organizational evolutionary patterns at the macro level of sector and organizational populations.

### Construction of the network

**Fig. 1:**
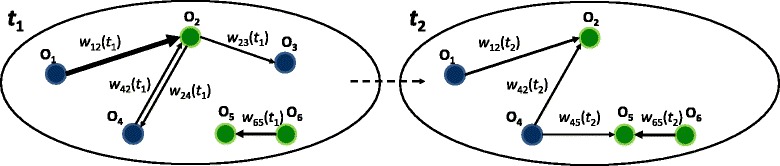
The evolving network of interorganizational labor flows. Organizations are represented by nodes in the network. Nodes have organizational-level properties: total amount of employees a given year, and belonging to the public or private sector. Employee movements between organizations are the links in the network, in this case directed edges. The flow *w*_*ij*_ represents the number of employees that moved from organization *i* to *j* that given year. Edges are weighted by their respective flows. All variables are a function of time and may change on a yearly basis

The labor flow network looks schematically as shown in Fig. 1. Each organization of 10 employees or more is represented by a node in the network. Nodes have two organization-level characteristics: its size and ownership sector (public or private). A link exists between two nodes at a given year if there has been movement between the corresponding organizations with respect to the previous year. Movements have direction; therefore, the links are directed edges. Edges can be reciprocal if there is movement from and to both nodes the same year. The number of employees moving constitutes the *flow*.

Edges are weighted by their respective flows, to account for the fact that the more people moving between two given organizations, the “closer” these organizations are and the higher the relative importance of that edge. Employees that stay in the organization from the previous year to the current year are represented by loops in the network, but we focus on the movements between organizations so loops are not considered. To sum up, the interorganizational network we study is directed— movements have an origin organization and a destination organization—, weighted, and temporal i.e. dynamic— organizations and movements may change over the years.

## Methods

### Network connectivity

As described in the Introduction, in order to study the connectivity and stability of organizational evolution, the population of organizations in Stockholm is modeled as a labor flow network where nodes are organizations and links are movements of people between organizations. In this network, connections between organizations take place through movements of people, either directly through a movement a given year, or indirectly through a sequence of movements that goes through several organizations, which is called a *directed path*.

Looking at the whole system a given year, there is in principle no reason to assume that all organizations will be connected to all others. There might be groups of organizations isolated from the rest. Those groups are called *components*. We distinguish between strongly- and weakly-connected components. A *strongly-connected component* (SCC) is a subset of the network such that there is at least one directed path from every node to all other nodes in the component. Organizations in the SCC can reach and be reached from each other. On the other hand, a *weakly-connected component* (WCC) is a subset of the network such that all nodes in it are connected, no matter the direction of the movement. This subset is less restrictive than the previous SCC case. We will work with the largest weakly-connected component WCC_1_, unless stated otherwise, in order to capture as much of the connected subset of the network as possible.

Secondly, we study the proportion of movements that take place within and between sectors through a sector mixing matrix. We use the *assortativity coefficient* *r*_sec_ (Newman 2003) to quantify the clustering of nodes along the sector dimension and to which extent this is the result of the relative amount of movements.

Organizations in the labor flow network are heterogeneous, and they might be more or less important according to different criteria. So thirdly, we consider centrality measures. The *degree* *k* of an organization is the number of movement edges that it has during a given year. Because our network is directed, we have an *in-degree* *k* ^in^ counting the edges coming into the organization and an *out-degree* *k* ^out^ counting the edges going out. The in-degree could offer some proxy for the attractiveness of an organization from the point of view of other organizations, as well as a recovery after a loss from previous years. For the out-degree, two possible interpretations are that high out-degree corresponds to an organization that has an active exchange of employees with the rest of the population, or to an organization that is downsizing or disappearing. Both in- and out-degrees should be analyzed together.

The most basic centrality measure is the *degree centrality*, which is simply the proportion of organizations that are connected to one organization; see for example (Freeman 1978) from the social network analysis (SNA) literature (Wasserman and Faust 1994). The higher the degree centrality, the better connected the organization is to the rest, in terms of incoming and outgoing movements. Next, for two organizations linked a given year, we define the distance as the inverse of the flow between them that year. The more people going from one organization to the other, the smaller the distance of the edge and viceversa. The distance of a directed path is the sum of the distances of the edges that constitute it. The path having the shortest distance is called a geodesic path. The *betweenness centrality* $B_{c}^{w}$ of an organization— see e.g. algorithms in (Brandes 2008) and (Freeman 1977) from the SNA literature— is the number of geodesic paths between all pairs of nodes that pass through a given node, as a fraction of all possible geodesic paths. Betweenness measures to what extent the given organization stands in between different parts of the network. A related measure is the *closeness centrality* $C_{c}^{w}$ of an organization, which is defined as the inverse of the average geodesic distance between that organization and the rest.

### Stability of labor flows

One important aspect of the evolution of an organizational population is given by the stability of the organizations that constitute the population. Consequently, we firstly look at differences in node survival by sector over time. Secondly, we look at statistical distributions of employee flows and its evolution in time. The sum of incoming (respectively outgoing) employees to an organization constitutes the *total flow* *F*, and in network science it is often called the strength of the node (Barrat et al. 2004). Thirdly, we focus on each individual movement edge, and perform a simple stability analysis to compute the likelihood of an edge to appear or disappear from one year to the next, conditioned on its weight.

Finally, the assortativity coefficient and its baseline for random link assignment is useful as a first approximation to control for imbalances in the relative abundance of movement edges within and between sectors. However, movements do not occur entirely at random; they are constrained by the very topology of the network, so that it is necessary to take some of those network properties into account. The most elementary properties are the edge weight and the degree heterogeneity. This is a form of local randomization, as opposed to global methods with local constrains like (Fagiolo et al. 2013).

The method we use here is called network backbone overlap analysis (Bajardi et al. 2011), and it works as follows. Each organization of total out-flow $F_{i}^{\text {out}}$ is connected to $k_{i}^{\text {out}}$ organizations, each having a flow of *w*_*ij*_ employees. Therefore, the normalized flow is $p_{ij}^{\text {out}}=w_{ij}/F_{i}^{\text {out}}$. The null hypothesis that serves as a baseline in this method is the random assignment of the normalized flows. The probability that a given normalized flow from organization *i* is randomly assigned to organization *j* is equivalent to the probability for that flow to not be randomly assigned to any of the $k_{i}^{\text {out}}-1$ other organizations to which organization *i* is linked, i.e. $\alpha _{ij}^{\text {out}}=(1-p_{ij}^{\text {out}})^{k_{i}^{\text {out}}-1}$. The same reasoning goes for the in-degrees and in-flows. An edge belongs to the network backbone a given year if $\alpha _{ij}^{\text {in}}<\alpha $ or $\alpha _{ij}^{\text {out}}<\alpha $, where alpha is a significance level^3^. This selection criterion is called the disparity filter method (Serrano et al. 2009). The *backbone overlap matrix* consists of all pairwise comparisons of network backbones at different years, and is given by the fraction of overlapping backbone edges. While the random baseline for the assortativity coefficient was a global one, the backbone overlap analysis uses a local fluctuation baseline.

All network measures were computed with networkx, a python package for the analysis of complex networks (Hagberg et al. 2008). Curve fitting was done with the python package powerlaw (Alstott et al. 2014).

## Results

### Network connectivity

#### Connected components

**Fig. 2:**
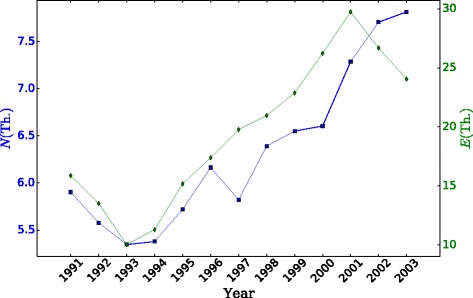
Time evolution of number of organizations (*N*, in thousands, left axis) and movement edges (*E*, in thousands, right axis) for the period 1991-2003

The evolution of the number of active organizations and movement edges on a yearly basis is shown in Fig. 2. Movements are based on a comparison of the given year with the previous one, and therefore all time series begin in 1991. Both time series follow a similar pattern, with a minimum of 5347 organizations and 10,042 movement edges in year 1993 during the crisis and a maximum of 7816 organizations in year 2003 and 29,738 edges in year 2000. The average over the whole time period is 6329 organizations and 19,513 movement edges. To note especially are the decrease in the number of organizations in 1997, and the decrease of edges with respect to nodes during the last two years.

But movement edges cluster the organizations in groups: the connected components. The number of strongly-connected components varies over time, but is on average close to 3574. The largest one of them, the largest strongly-connected component SCC_1_, concentrates on average $\phantom {\dot {i}\!}N_{\text {SCC}_{1}}=2749$ organizations and $\phantom {\dot {i}\!}E_{\text {SCC}_{1}}=\text {15,540}$ movement edges, that is to say, an average of 43% of the organizations and 80% of the movements. Out of an average of 1206 weakly-connected components, the largest weakly-connected component WCC_1_ concentrates $\phantom {\dot {i}\!}N_{\text {WCC}_{1}}=5074$ organizations and $\phantom {\dot {i}\!}E_{\text {WCC}_{1}}=\text {19,462}$ movement edges, closer to the average figures for the whole network, with almost all edges included. The rest of the components are made of either isolated nodes— i.e. organizations that do not exchange people with other organizations— or very small configurations like groups of two or three organizations. Concretely, this means that during any given year, one could reach most of the organizations of 10 employees or more by following the interorganizational movements during that year, although ignoring the direction of the movements. Processes that are channeled through networks can spread out at a faster pace than if the system were composed of clusters of comparable size isolated from each other.

**Fig. 3:**
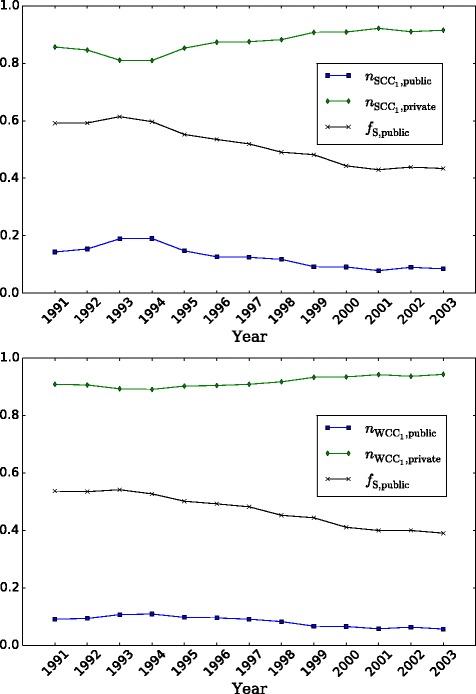
Sector composition as fraction of active number of organizations, by largest component. The plots show the fractions *n* of organizations by sector, accompanied by the fraction *f*_*S*_ of employees in the public sector with respect to the total number of employees in the population. Upper panel: composition in largest strongly-connected component SCC_1_. Lower panel: composition in largest weakly-connected component WCC_1_

Looking at sector-specific network properties, we plot the time evolution of the number of active organizations by sector as a fraction *n* of the total number of active nodes in Fig. 3. The largest strongly-connected component is shown in the upper panel. The plot also shows the fraction *f*_*S*_ of employees working in the public sector. We see that there is an increase in relative terms of public-sector nodes due to the inactivation of private organizations during the crisis period 1991-1993. After 1999 the fractions are roughly stable in time, with around 10% of the nodes in the public sector, and 90% in the private. Furthermore, the fractions of employees and organizations in the public sector are very different in magnitude (although they exhibit similar trends). Between 60% and 40% of the employees in this component work for the public sector and are concentrated in around 10% of the organizations.

The largest weakly-connected component is shown in the lower panel. Here we see that the impact of the first years is attenuated with respect to the SCC_1_ case, and the stable values after 1999 are now 6% in the public sector and 94% percent in the private sector. The fraction of employees working in the public sector is overall lower than in the case of *S* *C* *C*_1_, meaning that switching from strongly- to weakly-connected component adds private sector employees to the picture. That is to say, the WCC_1_ case covers all nodes connected to the largest component, which includes a core of more tightly connected nodes that is the SCC_1_. On the one hand, the fact that the fraction of public nodes is smaller in WCC_1_ compared to SCC_1_ means that the difference between the two components is made out of mainly private nodes. We know that the WCC_1_ has 1.6-2.6 times more nodes than the SCC_1_. Thus, the weakly-connected component has a large periphery of less connected private organizations that cannot reach the core, for instance because they only have incoming movements or only outgoing. On the other hand, the decline of private nodes in SCC_1_ during the crisis years shows that in that period even the well-connected private nodes were leaving the system.

**Fig. 4:**
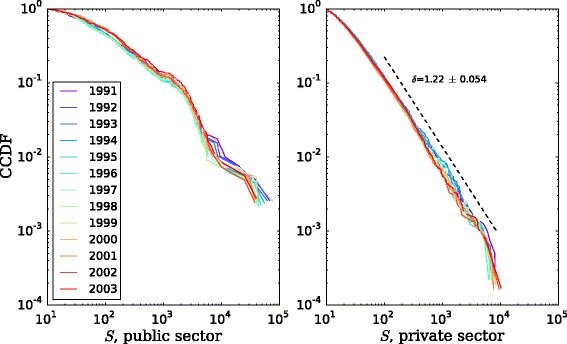
Size distribution of the nodes in the largest weakly-connected component, by year and sector. Average power-law maximum likelihood estimation (MLE) fit (dashed line) and exponent *δ* shown for the private sector

The nodes in the network are not homogeneous in their connectivity, but neither are they with respect to their size. In Fig. 4 we show the size distribution for the nodes in the largest weakly-connected component, by year and ownership sector. Both distributions are skewed, indicating that there are very few organizations with a large number of employees, but many organizations employing a relatively small number of employees. The patterns are quite stable over time, and in particular, the statistical pattern for private organizations follows a power-law distribution scaling as ∝*S* ^−*δ*^, with the value of the exponent *δ* in the range observed for similar systems of organizations in other contexts, e.g. (Axtell 2001).

#### Sector assortativity

**Fig. 5:**
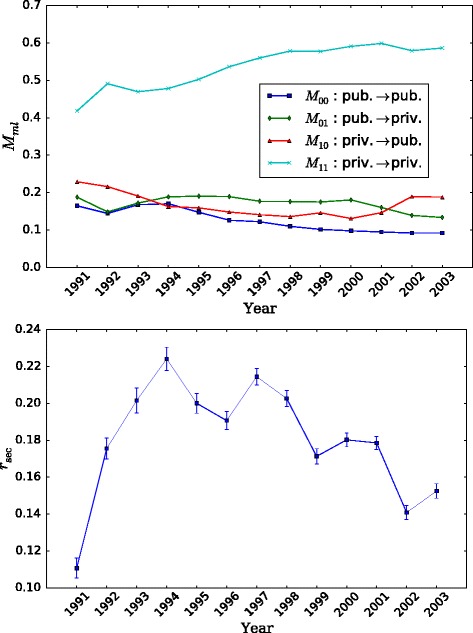
Time evolution of sector mixing indicators. Upper panel: elements of the sector mixing matrix: fraction of movement edges *M*_*ml*_ from sector *m* to sector *l*. Lower panel: sector assortativity coefficient *r*_sec_ to determine whether the observed edge mixing pattern is just due to random movements between to groups of unbalanced number of nodes

In order to get a sense of the relative prevalence of edges connecting the sectors, a complementary picture is provided in Fig. 5, upper panel, where we plot the sector mixing matrix [*M*_*ml*_] for the weakly-connected component. A matrix element *M*_*ml*_ is the fraction of movements edges that go from sector *m* to sector *l*. We observe a bottom fraction of movements within the public sector. The fraction goes up in 1993-94 and then slightly declines over time, in favor of the rest, a trend similar to the evolution of the fraction of employees in the public sector. After a decay in 1993, movement edges within the private sector (topmost curve) increase in relative abundance, from 40 to 60%. In the movement interface between sectors, the fraction of edges going from private to public declines, stays relatively stable for most of the period and goes up the last 3 years. Contrary to that, the fraction of public-to-private edges goes up, stays stable for most years in the middle and then declines.

But as we have seen before (Fig. 3, lower panel) there are many more organizations within the private sector than the private one. Consequently, even if the same amount of movements happened at random, people would most likely start in the private sector and move to another organization within it, or if they start in the public sector, they would move to the private. So we need to quantitatively assess whether this sector mixing could be due to pure randomness in the distribution of edges on top of an asymmetric node distribution. We compute the assortativity coefficient by sector *r*_sec_ for each year and plot the result in Fig. 5, lower panel. The error bars are computed according to the expression for error in intraclass correlations, Eq. (5) in Newman (2003). The coefficient can be interpreted as a Pearson correlation coefficient, and we see that there is positive assortativity, namely that organizations of the same sector tend to be connected to a greater extent than what is expected by chance (*r*_sec_=0). A positive coefficient value is typical of many complex social networks with values in the same order we get here, see Newman (2003) for a table of typical assortativity values.

We also note that there is a peak of *r*_sec_ and then declining trend after 1994, consistent with the decrease in public-to-public edges in that period. Additionally, if we recall from Fig. 2 there is a decrease in the total number of movement edges towards the end of the period, and we can relate this to the decrease in the fraction of movements edges *M*_01_ from the public to the private sector, in spite of the decreasing tendency in assortativity for those years.

**Fig. 6:**
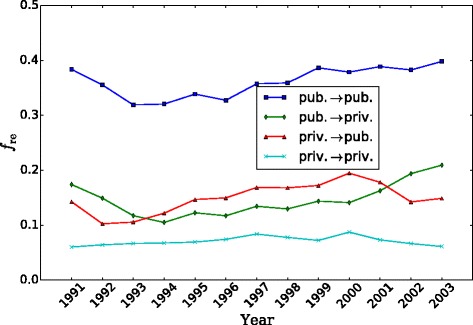
Time evolution of the fraction of reciprocated edges by link type. A reciprocated edge is an edge where there is movement of employees on both directions during the same year

Finally, reciprocity in movement edges offers an additional indication that there is a closer exchange between organizations. We plot in Fig. 6 the fraction of all edges in a given link type (e.g. from public to private sector) that are reciprocated during the same year. Edges within the public sectors are highly reciprocated. The time evolution follows the global trend we saw for nodes and edges (Fig. 2). The lowest fraction corresponds to movement edges within the private sector, but here it is essentially constant in time. The interfaces between sectors have intermediate fraction values, with private-to-public edges more highly reciprocated during the recovery years.

#### Centrality distributions

**Fig. 7:**
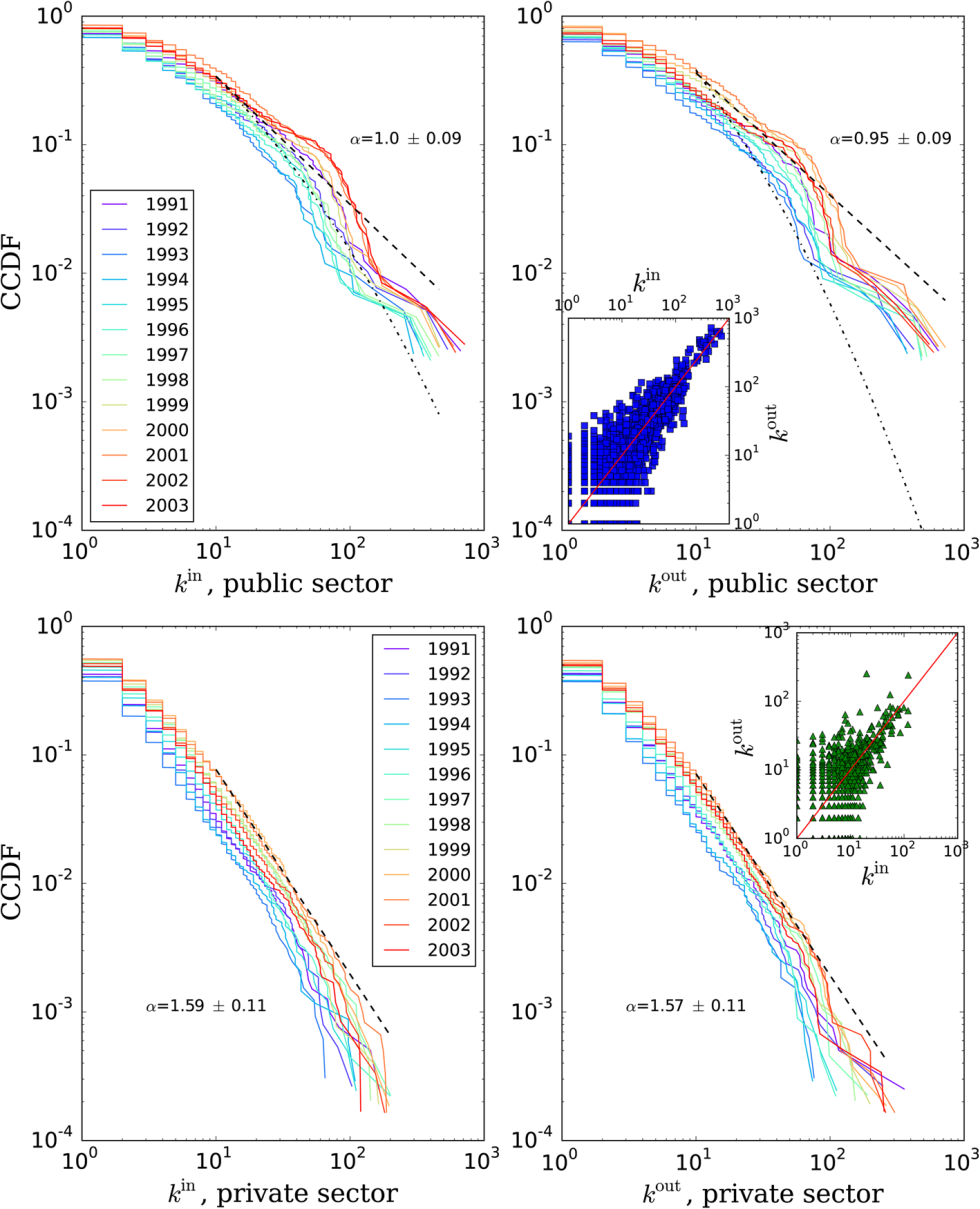
In-/out-degree distributions, by year and sector. The in-degree of a node is the number of incoming movement edges regardless of the number of employees moving, and similarly the out-degree is the number of outgoing movement edges. Average power-law maximum likelihood estimation (MLE) fits (dashed line) and exponents *α* shown, starting at degree 10. For the public sector, the corresponding MLE fits to a lognormal distribution (dotted line) are shown. The insets present the correlation between in- and out-degree, with the identity line for reference

As we have seen in the “Methods” section, an aspect of network connectivity is the distribution of in- and out-degrees. We can see in Fig. 7 that the shape of the cumulative distribution is similar for in- and out-degree. The distributions for the private sector follow a power law of the form *P*(*K*≥*k*)∼*k* ^−*α*^. The exponents *α* are similar for both in- and out-degree distributions. The implications of this distribution are that the degree is very non-uniformly distributed across organizations: the largest fraction of organizations has degrees below 10, while very few have extremely high degree. The distribution does not present substantial discontinuities, meaning that there are organizations having degrees at all orders of magnitude, a statistical pattern also observed for the size distribution (Axtell 2001).

The public sector on the other hand exhibits consistently fatter tails whose shape is somehow closer to a lognormal distribution. The presence of a public sector lognormal degree distribution as opposed to a private sector power-law degree distribution may be an indication of two competing evolutionary logics as advanced in the introduction. In other context like financial markets and statistics of wealth distribution, it has been argued that the low-end segment of the economy works in an additive way (paying for exactly the amount one gets indebted for), while the high-end segment works through investment, which is a multiplicative process. This fact results in the former wealth distribution being exponential and the latter being a power law (Yakovenko and Barkley Rosser 2009). Patterns of degree assortativity can be studied as well beyond the simple first-order degree correlation, like the nearest-neighbor degree correlations in Guerrero and Axtell (2013).

**Fig. 8:**
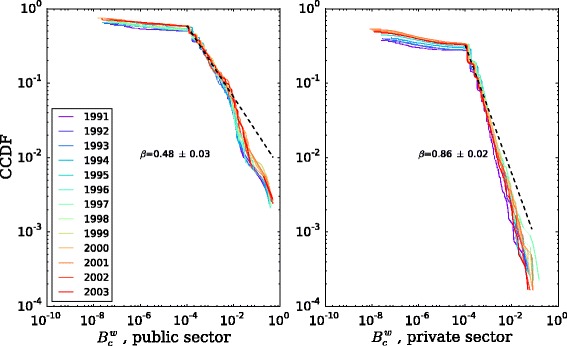
Betweenness centrality distributions, by year and sector. Betweenness is a node-level measure of the fraction of paths between organizations that go through a given organization. It takes into account the direction of the edges and is weighted by the flow of the movement edges. Average power-law MLE fits and exponents *β* shown, starting at 10^−4^

For the weighted betweenness centrality $B_{c,i}^{w}$, we plot the cumulative distribution for every year in Fig. 8, by sector. We observe once again a stable statistical pattern within each sector. Moreover, we can identify two regimes: in the three orders of magnitude below $B_{c}^{w}=10^{-4}$ there is very little change in the fraction of nodes, and above that value the distributions are, like in the case of the degree, heavily skewed and with a power-law-like tail of the form $P\left (b_{c}^{w}\geq B_{c}^{w}\right)\sim k^{-\beta }$. This means that there are a lot of organizations with very low betweenness centrality scores, a few less with higher, and just some very few with very high values. Recalling the definition, this implies that the network, for all years, has very few key nodes through which many movement paths go through, connecting different parts of the network. Regarding sector, the distributions have qualitatively the same shape, with the public distribution decaying slower than the private one.

### Stability of labor flows

#### Node stability and the effect of organizational size

**Fig. 9:**
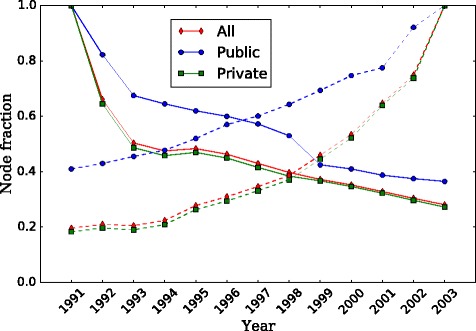
Node stability over time, by sector. Fraction of initial organizations still surviving in subsequent years (forward in time, solid line), and fraction the organizations at the last year present in previous years (backwards in time, dashed line)

A node stability analysis is shown in Fig. 9. The solid lines show the fraction of initial nodes that continue active during the period, and the dashed line shows the analogous but backwards in time from 2003. Around 40% of the public organizations that existed in 1991 exist in 2003, and roughly the same is true backwards. Less than 30% of private organizations survive through the period, and less than 20% of the organizations active in 2003 were active at the beginning of the 1990s. Global fractions are close to the private trends due to the higher prevalence of private organizations.

**Fig. 10:**
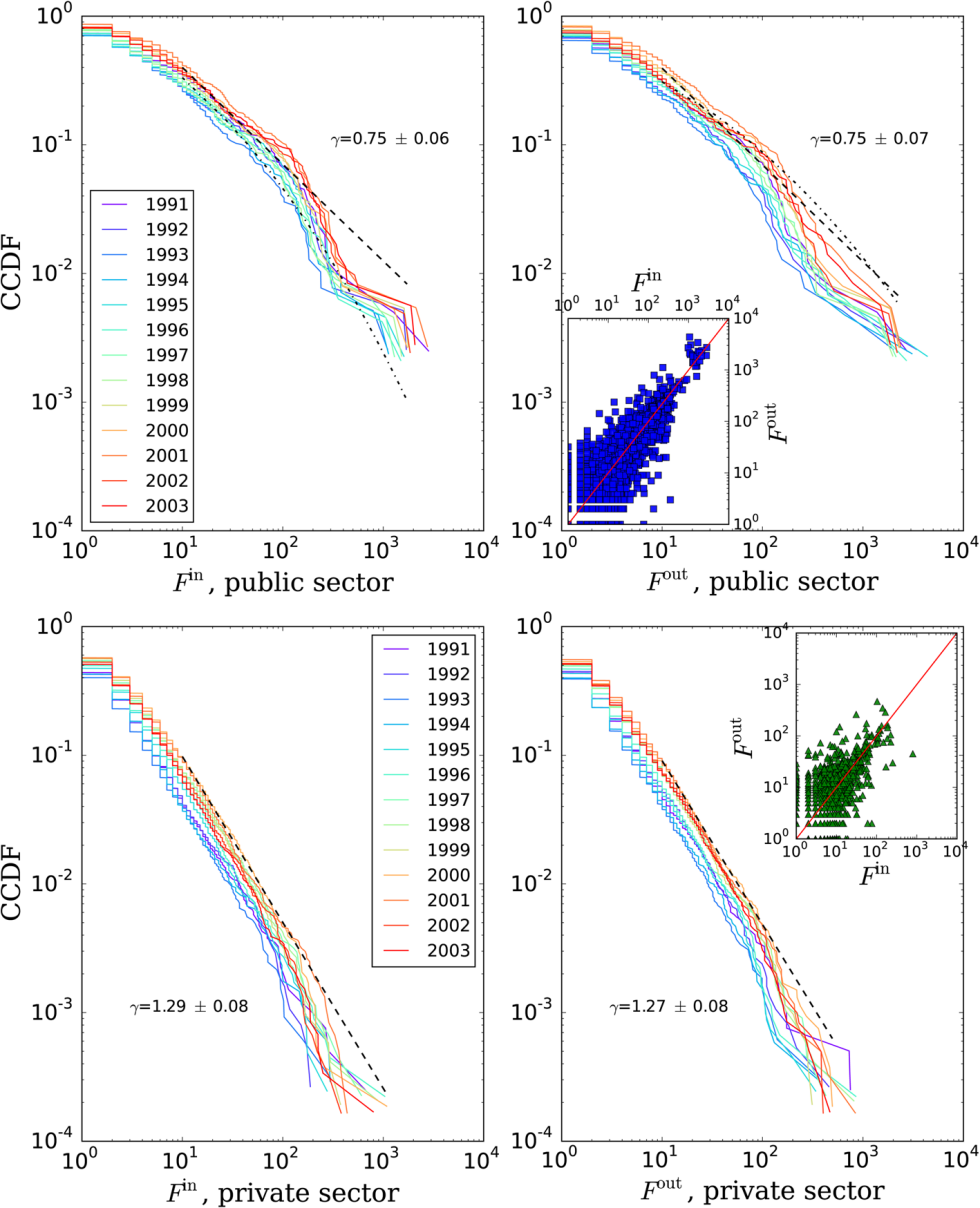
Incoming and outgoing total flow distributions, by year and sector. They are the weighted analogues of the in- and out-degree distributions in Fig. 7. Average power-law Maximum likelihood fits (dashed line) and exponents *γ* shown, starting at degree 10. For the public sector, the corresponding MLE fits to a lognormal distribution (dotted line) are shown. The insets present the correlation between incoming and outgoing flows, with the identity line for reference

We have seen that the number of movement edges an organization received, i.e. the in-degree, is highly correlated to the number of edges it sends out, i.e. the out-degree. Comparing the public and private sectors for the flow distributions (Fig. 10) we observe a pattern similar to the degree distributions. The exponents *γ* are smaller in this case, meaning that the distribution decays more slowly in the latter case.

Moreover, the size of an organization a given year is the size the previous year plus the incoming flow and minus the outgoing during the course of year. Size is likely to be correlated with movement edges and with flows. Large organizations mobilize more resources, and can be engaged in more diverse activities with many other organizations, resulting in more movements and larger employee flows (Saito et al. 2007). Furthermore, in their book on organizational demography, Carroll and Hannan (2000, ch.14) state that organizational size is the main predictor variable for relevant organizational outcomes such as rates of founding and disbanding of organizations.

**Fig. 11:**
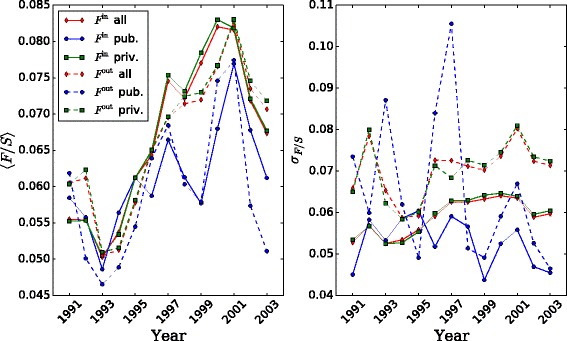
Time evolution of mean and standard deviation of in- and out-flow divided by organizational size, by sector

Therefore, part of the observed patterns must be due to size effects. Dividing the flow by the size of the organization that year gives the number of employees that moved, as a fraction of the employees that year; see Fig. 11. We observe that the normalization by size puts the average flows for the different sectors closer to the time series for the whole population in Fig. 2. There is a general trend towards higher average values as time elapses. The private sector has higher average flows. There is a negative peak in 1993 corresponding to the worst of the crisis years, and a positive peak mainly in 2001. The standard deviation pattern is not as homogeneous as the average pattern. The pattern for public sector fluctuates much more than the time series for the total population, with high deviation in 1993, high in 1997 and in 2001.

As another aspect of size effects, it is important to understand how these network properties change with organizational size. Looking at size scaling is complementary to other analyses, and its use is more common in disciplines outside the social sciences. For example, within biology, there are classic works such as the one by Thompson (1992) that examine how various physiological quantities scale with an animal’s size. Scaling is an established technique in statistical physics, but also in the study of complex systems, as is the case of the scaling of city properties (innovation, production output) with its size (Bettencourt et al. 2007).

**Fig. 12:**
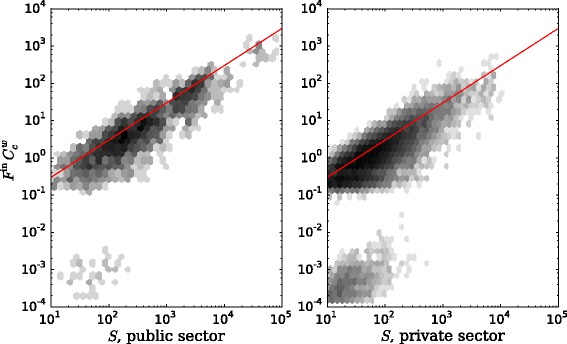
Size scaling of incoming flow weighted by closeness centrality, by sector. Organizations with betweenness centrality scores larger than 10^−4^. All years pooled together. Hexagonal bin plot colored by point density, black corresponding to highest point density

We look into the scaling of incoming flows with organizational size. Outgoing flows, as seen before, are highly correlated to incoming flows, so the results are qualitatively similar. It has been shown that larger firms have more connections and the degree scales as a power law (Guerrero and Axtell 2013; Saito et al. 2007). We use here what we have learned about node properties in this network, and weight the nodes by multiplying each incoming flow by its closeness centrality score. The size scaling for the incoming flow is shown in Fig. 12. We have also used the betweenness centrality scores to distinguish between the two regimes: organizations with betweenness below or equal to 10^−4^ and organizations with betweenness above 10^−4^. The limit is set based on information from Fig. 8. We see that organizations split into two clear groups: some have low weighted flow and some have high weighted flow. The group of low weighted flow is composed mainly of small-sized organizations between 10 and 100 employees. This means that there is a group of small organizations with very low average distance to the rest of the network.

There is a significant difference with the group of high weighted incoming flow. Weighted flows scale on average as a power law of organizational size. Interestingly, small-sized organizations are present in all these cases, but only medium- and large-sized ones have high closeness. This indicates that most likely the big and medium organizations sit at the center of the network, receiving and sending many employees, with outer layers of the small organizations exchanging very few people with the rest and being further away in network distance.

#### Movement stability and network backbone overlap analysis

**Fig. 13:**
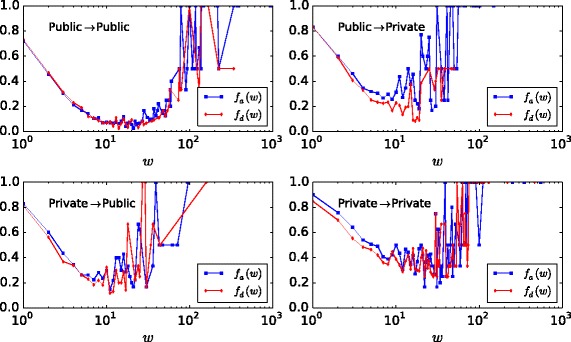
Movement stability analysis. Fraction of appearing and disappearing movement links— *f*_*a*_,*f*_*d*_ respectively— as a function of the edge weight *w* (employee flow), by link type. All years pooled together

The movement stability analysis by link type is shown in Fig. 13. We see that the fraction of appearing edges *f*_*a*_ is in general slightly higher than the fraction of disappearing edges *f*_*d*_. Low and high edge weight represent infrequently small/large movements, and thus fluctuate more than middleweight movement links. Movements within the public sector have lower change fractions and are therefore more stable; the opposite is true for links within the private sector. The interfaces between sectors constitute an intermediate and almost symmetrical case, with higher fraction of public-to-private appearing edges. A similar pattern has been obtained in a seemingly very different kind of flow network: the movement of cattle between farms and trade centers (Bajardi et al. 2011).

**Fig. 14:**
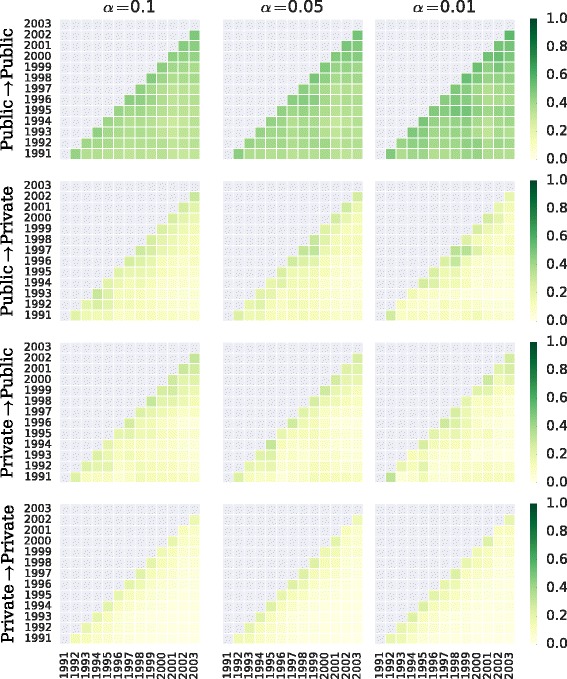
Network backbone overlap matrix. Fraction of overlapping links between the backbone networks of two given years, by link type and evaluated at different significance levels *α* by disparity filter method

Since size and degree are on average proportional; this we know from the scaling relation in Fig. 12. Thus, controlling for node degree means also on average to take into account the organizational size. We compute the network backbone of the labor flow network for each year and link type, evaluated at different significance levels (Bajardi et al. 2011). The backbone overlap matrix comes in Fig. 14. The lower the *α* parameter, the more demanding the selection condition, thus the more restrictive the network backbone and the higher the overlap. We see that movement edges within the public sector are more stable, as the backbone of this network subset shows high overlap for most years and the highest among link types at all *α* parameters (on average between 48% and 56%). This is not the case of backbone of movements within private sector organizations, which features lower (on average 22 to 24%) and faster decaying overlap. The two interface backbones constitute intermediate cases. Importantly, an edge belonging to the backbone is correlated with specific node properties. For instance, at high *α*=0.10, the majority of backbone edges are not reciprocated, while the opposite is true for *α*=0.01. Edges between large organizations are progressively more represented in the backbone as *α* decreases.

**Fig. 15:**
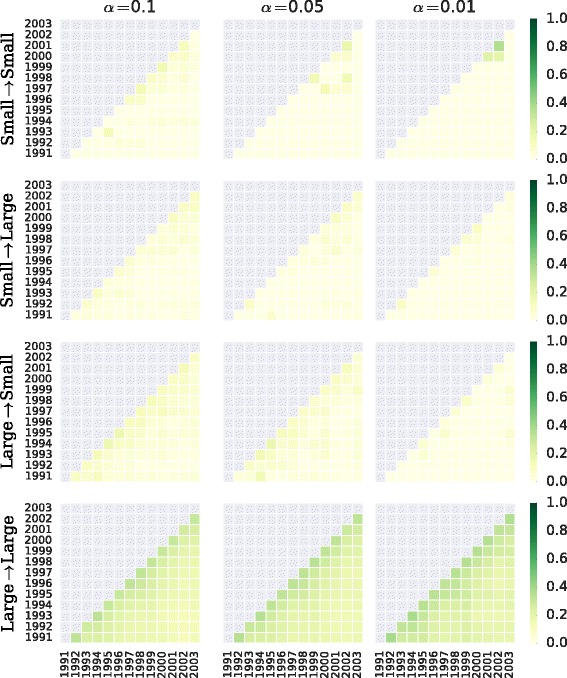
Network backbone overlap matrix. Same logic as in Fig. 14, but now broken down by size range: small (up to 100 employees) and large (more than 100 employees)

However, there are some considerations to this analysis, that have to do with the differences in the distribution of node properties. Due to the skewness in the size distribution (see Fig. 4) and the fact that public organizations tend to be larger than private organizations, the backbone has a size dependence. Thus, in Fig. 15 we have partitioned the network backbones by size ranges: organizations with up to 100 employees on the one hand, and organizations with more than 100 employees on the other hand. We can see that the overlap is clearly higher for backbone edges within the public sector.

**Fig. 16:**
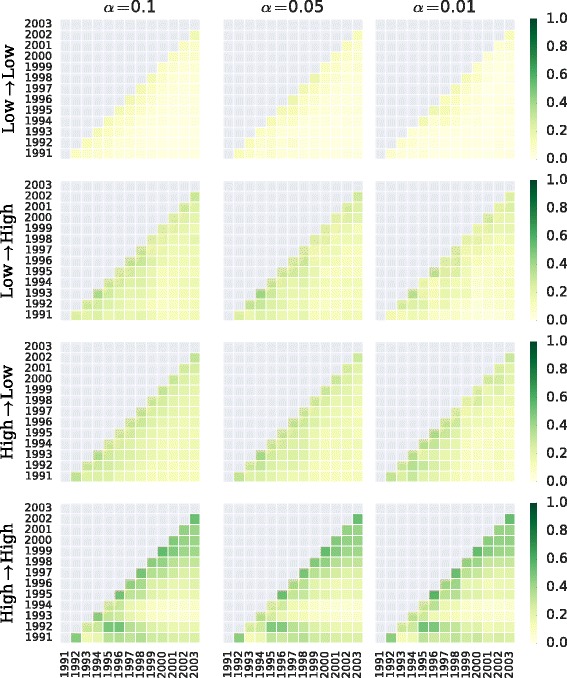
Network backbone overlap matrix. Same logic as in Fig. 14, but now broken down by total degree range (i.e. the sum of the in- and out-degrees): low (up to 100 movement edges) and large (more than 100 movement edges)

As we saw in the scaling analysis, size and degree are correlated, and the degree distribution is skewed as well (Fig. 7). So, a similar analysis but for ranges based on the total degree (i.e. the sum of the in- and out-degrees) is shown in Fig. 16. The total degree ranges are up to 100 links, and more than a 100 links. Here, we still observe that the most stable overlapping network backbones take place among edges between organizations with high total degree. The overlap is also relatively high for the other edges involving an organization with high total degree, although the correlation does not stretch as far in time as for the previous case.

## Discussion

In this article, we have used the network of interorganizational labor flows and tools and concepts from network science to inform the study of organizational evolution at the level of sector dynamics, in particular along the dimensions of connectivity and stability of labor flow patterns. We have used Swedish register data on employment in the Stockholm Region to study the evolution in the network of interorganizational employee flows. These longitudinal data provide great opportunities for research on organizational dynamics, as it allows to examine how individual movements generate the emerging statistical properties of a whole population of organizations, and to get insights into differences in the dynamics of the public and private sectors.

Describing the organizational population through the evolutionary approach to organizational change (Aldrich and Ruef 2006), we focused primarily on sector differences and similarities, in particular regarding connectivity and stability. More analyses into the other four processes is needed, for instance regarding proxies for competition between sectors, called struggle in the evolutionary approach used here (Aldrich and Ruef 2006).

Methodologically, network analysis can reach conclusions about interaction that classical studies in organizational evolution using mainly regression analysis— as in the organizational ecological approach (Hannan and Freeman 1989)— cannot show. Regarding network connectivity, most of the organizations and practically all of the movements belong to the largest weakly-connected component *W* *C* *C*_1_, and as such are connected through movement edges. The number of incoming movements edges (the in-degree) is positively and highly correlated with the number of outgoing edges (the out-degree) and the same is true for incoming and outgoing flows. There is positive sector assortativity, indicating that movements within sectors are more prevalent than across sectors; the value is over the one expected by mere random movements.

Some statistical patterns are consistent regardless of the sector. Degree distributions are skewed and “fat”-tailed, the overall pattern closer to a power law for the private sector and between a lognormal and a power law for the public sector. This implies that in both cases there are very few organizations that are extremely central in terms of the number of organizations they are connected to. Many organizations have very low degree, but at the same time, one finds some medium-range organizations with intermediate degree values. The distribution of betweenness centrality is also skewed and heavy-tailed, indicating huge concentration on edges and brokerage importance in very few organizations, while there is a regime of low betweenness centrality spanning several orders of magnitude.

Sectors differ in other non-trivial respects. The public sector is relatively more tightly connected than the private one, in the sense of having more presence in the strongly-connected relative to the weakly-connected component and higher fraction of reciprocated edges. Stability varies across ownership sector. Public organizations significantly are more stable than private ones. The movements within the public sector are also more stable, and the network backbone of these movements exhibits much higher overlap over time. The private sector on the other hand has less stable organizations and employee flows. When taking into account differences in node size and total degree, we see that the most stable backbone movement edges exist between large organizations with relatively high total degree.

Additionally, the public sector exhibits consistently fatter tails than the private one in all distributions. Flows of people between organizations differ as well across sectors. When dividing by size, we see that the flow time series gets closer to the overall evolution of the network, which indicates that flow statistics are strongly driven by size effects. We further analyzed this size dependence of flows in combination with the closeness and betweenness distributions, and find that medium and large-sized organizations have high closeness and exhibit a power-law scaling of incoming flows with size, together with some small-sized organizations. If the sectors have indeed two different logics and respond differently to external shocks like crises, both in their connectivity and their network stability, we can speculate about policy implications. For instance, this could be relevant in relation to debates on privatization of previously public activities like education and health. On the managerial side, questions on efficiency and productivity could be formulated in relation to the interactions between organizations and their stability, helping to define better management indicators and informing the decision-making process.

We should say few words about considering movements on a yearly basis. Our reflections on connectivity and paths, as we mentioned, are based global measures encompassing the whole network. Because the network is constructed on yearly employment movements, there is a limit on how far we can move in a year following these edges. This will depend largely on which process one theorizes is being channeled through the network. For instance, if job mobility through this network is the question of interest, then some of our longest movement paths might simply not be feasible, because a year could give enough time to go through them all. However, if the question is about finding information about jobs through the network. This is especially important to keep in mind for the network measures involving movement paths, like closeness and betweenness centrality.

Throughout this paper, we have been mainly concerned with using the network paradigm to perform organizational evolution analyses based on employee movements. Some additional analyses can be performed by relaxing one or more of the simplifications we took in building the network. A flow analysis considering the people entering/leaving the Region and the ones staying in the organizations (i.e. the loops we removed) is one such alternative. Further analyses of this temporal network treatment are also possible, e.g. concerning the study of organizational inertial properties, network searchability, cascading effects, etc. (Holme and Saramäki 2013). One could incorporate additional node properties as well, in this case for instance workplace industrial activity. This would allow for the study of flows within and between industries. Also, there is a battery of algorithms for finding communities in networks; see Fortunato (2010) for a recent review. An interesting avenue to pursue would be to correlate such communities emerging from the network connectivity with existing node properties such as sector and industry.

Some of the unused variables can be incorporated in the network analysis, typically the industrial activity. This could even enrich the picture of the public and private sectors, because activities, as mentioned in the Introduction, are clustered by ownership sector. A whole new range of network-related analysis is possible along these lines, for example community structure analysis of industry-specific trade patterns (Cingolani et al. 2015). The interplay between the geographical dimension and the labor flows is also of interest, as it has been shown that many relations decay with geographical distance (Butts et al. 2012).

Another interesting point has to do with the connection between organizational growth distributions and the distributions of network properties. Yet one more dimension to study could be to distinguish between profit and non-profit activities. This kind of studies have increasingly interested sociologists and are worth of further exploration (DiMaggio and Anheier 1990). Moreover, economic studies show that the willingness to change jobs are significantly affected when we consider this dimension (Becchetti et al. 2013).

So far, we have looked at the problem mainly through the point of view of the whole population of organizations. Given that the data allow for it, we can take an alternative point of view and think of the system as a combination of individual trajectories in time, each trajectory consisting of the time series of an individual’s membership career. From this trajectory point of view, one could study individual permanence and probability to switch jobs, conditioned on organizational properties such as size, sector and industry. Such an approach has been applied to different research domains, e.g. the transition from education to employment (Scherer 2001), and is furthermore the subject of an ongoing study on the same database.

On the modeling side, computational social science has emerged as a combination of analyses of big-data and large-scale simulation (Conte et al. 2012), and one aspect (not the only one, though) takes in the form of agent-based models searching for generative explanations of macro-phenomena based on micro-level individual interactions (Epstein 2014). Using networks based on empirical data to calibrate such agent-based models is seen by some social scientists as a cornerstone for the development and testing of analytical sociological theories (Hedström 2005). Other possible and somehow related research avenues have to do with developments in economic network agent-based modeling. Following up on the research referred to here in Guerrero and Axtell (2013), Lopez et al. (2015) propose a modeling framework where economic agents change jobs or stay in the firm, their decision conditioned on a number of parameters representing information on job availability, as well as on the structure of the firm network.

## Endnotes

^1^ Note that this analytical dimension should not be confused with the network measure ‘connectivity’, which is the number of independent paths between two given nodes (Newman 2010, p.147).

^2^ The name of the full database is LISA, for Longitudinal integration database for health insurance and labour market studies. More information on the source of the data can be found at the Statistics Sweden website: http://www.scb.se/lisa-en/.

^3^ This parameter should not be confused with a significance level in a statistical inference context.
